# Catalytic Reaction Model of Suicide

**DOI:** 10.3389/fpsyt.2022.817224

**Published:** 2022-03-09

**Authors:** Pamela McPherson, Saveen Sall, Aurianna Santos, Willie Thompson, Donard S. Dwyer

**Affiliations:** ^1^Department of Psychiatry and Behavioral Medicine, Shreveport, LA, United States; ^2^Department of Pharmacology, Toxicology and Neuroscience, LSU Health Shreveport, Shreveport, LA, United States

**Keywords:** suicide, suicidal ideation (SI), threat assessment, diminished motivation, impulsivity

## Abstract

Suicide is a devastating outcome of unresolved issues that affect mental health, general wellbeing and socioeconomic stress. The biology of suicidal behavior is still poorly understood, although progress has been made. Suicidal behavior runs in families and genetic studies have provided initial glimpses into potential genes that contribute to suicide risk. Here, we attempt to unify the biology and behavioral dimensions into a model that can guide research in this area. The proposed model envisions suicidal behavior as a catalytic reaction that may result in suicide depending on the conditions, analogously to enzyme catalysis of chemical reactions. A wide array of substrates or reactants, such as hopelessness, depression, debilitating illnesses and diminished motivation can mobilize suicidal thoughts and behaviors (STBs), which can then catalyze the final step/act of suicide. Here, we focus on three biological substrates in particular: threat assessment, motivation to engage in life and impulsivity. Genetic risk factors can affect each of these processes and tilt the balance toward suicidal behavior when existential crises (real or perceived) emerge such as loss of a loved one, sudden changes in social status or serious health issues. Although suicide is a uniquely human behavior, many of the fundamental biological processes are evolutionarily conserved. Insights from animal models may help to shape our understanding of suicidal behavior in man. By examining counterparts of the major biological processes in other organisms, new ideas about the role of genetic risk factors may emerge along with possible therapeutic interventions or preventive measures.

The anguish of someone preparing to take their own life is clearly unbearable and staggers comprehension. Moreover, the loss and grief of loved ones, family members and colleagues of people who die by suicide are incalculable and unrelenting. Unfortunately, suicides and suicide attempts are not rare, isolated events. Worldwide more than 700,000 people die by suicide each year and about 20 times this number will attempt suicide (1). Depending on the age group, suicide is a leading cause of death (1). Beyond the act of suicide or attempted self-harm, suicidal thoughts and behaviors (STBs) manifest in about 10% of the population at some time in their life (2) with higher prevalence in those with psychiatric disorders such as major depression, bipolar disorder, schizophrenia and anxiety disorders (3). In fact, the majority (~90%) of individuals who die by suicide suffered from a psychiatric condition prior to taking their life (4). However, mental illness is not a required state of mind and it is counterproductive to believe this is the case. Any theory must consider suicide broadly and clinicians must screen for suicidal ideations in addition to psychiatric disorders.

Suicide is potentially a preventable outcome if we can identify individuals with plans and intentions prior to their final actions. Although progress has been made in identifying risk factors for suicide such as presence of psychiatric disorders, previous suicide or self-harm episodes and advanced suicidal ideation, we still lack precise prediction methods. Furthermore, the success of available interventions is limited.

The fact that suicide tends to cluster in families (5, 6) provides a clue that genetic predisposition may be an important factor. Indeed, the heritability of suicidal behavior is on the order of 40–50% (7, 8), commensurate with the heritability of psychiatric disorders including major depression and bipolar disorder (9–11). Genome-wide association studies (GWAS) and other investigations have identified gene candidates that increase the risk for STBs and suicide (12–16); however, none of the risk genes have yet reached the stage of predictive value. This failure owes, in part, to the lack of context or biological mechanisms that link the risk genes to behaviors associated with suicide.

The neurobiological basis of suicidal behavior is multifaceted and includes significant contributions from the serotonergic system, the hypothalamic-pituitary-adrenal (HPA) axis [(17); discussed below], neuroimmune function and the endocrine system. Excellent reviews on these topics are available (17–22). Cytokines, especially IL-6 and TGF-b, appear to play a role in the emergence of STBs (14, 20). In addition to the HPA axis, neuroendocrine factors such as neuropeptide Y [NPY; (23–25)] and insulin (26, 27) have been implicated in the emergence of suicidal behavior, which will be discussed further in a later section. Detailed discussion of the neurobiology of suicide is beyond the scope of this review.

The purpose of this article is to introduce a novel model of suicide that integrates biology, genetics and brain circuitry to explain the various factors driving STBs. We will begin by describing current major theories of suicide and discussing their shortcomings before developing the catalytic reaction model of suicide.

## Theories of Suicide

To understand the factors contributing to suicidal behavior and suicide, different theories, dealing mainly with psychological mediators, have been postulated. Most recent models fall into the broad classification of ideation-to-action theories (28) and will be briefly outlined here as a basis for comparison with the new catalytic reaction model, which redefines this ongoing process.

Mann and colleagues (29) proposed a stress-diathesis model of suicide based on differences observed in state and trait factors between suicide attempters and matched psychiatric patients without suicide attempts. Although not explicitly an ideation-to-action theory, the model does treat the suicidal act as a separate event. Risk factors that distinguished persons attempting suicide in this study included suicidal ideation, hopelessness, impulsivity and fewer reasons for living along with a family history of suicidal acts, head injury, smoking and childhood abuse. The suicidal act may be precipitated by a combination of baseline stress or a psychiatric disorder coupled with feelings of hopelessness or new perceived threats, which trigger suicidal ideation. The switch from risk state to action may be hastened by impulsive behavior that facilitates acting on suicidal thoughts.

The Interpersonal Theory of suicide was formulated by Joiner et al. (30) and was the first to specify two distinct stages to suicide: the emergence of suicidal desire and the capability to engage in suicidal behavior. Two main factors drive suicidal desire, namely thwarted belongingness and perceived burdensomeness. In addition, there may be a sense of hopelessness about these two emotional states. The capability for suicide stems from adaptation to painful events such as family conflict, abuse, mental disorders, physical illness and previous suicide attempts that reduces the fear of death and disinhibits decisions for lethal action. Finally, the desire for suicide is a dynamic process, whereas the capability for suicide is thought to reflect stable and unchanging factors.

O'Connor developed the Integrated Motivational-Volitional Model of suicide in a series of papers (31, 32). Similar to Joiner's theory, there are two behavioral stages – the motivation to commit suicide and the volition or commitment to carry out lethal action – that are distinct and governed by different factors. The motivational phase is driven by feelings of defeat/humiliation and entrapment leading to suicidal ideation and initial intent. Separately, the ultimate decision to end one's life is moderated by such factors as impulsivity, fearlessness about death and cognitive input (planning) along with access to lethal means. In addition, O'Connor proposed a pre-motivational phase that included extant risk factors (e.g., genetic or cognitive vulnerability), environmental conditions and stressful life events, which together can determine the propensity for STBs.

To illustrate the range of conceptualization of possible causes of suicide, we briefly mention three additional theories that are variations on the ideation-to-action scheme, namely Three-Step Theory (33) Fluid Vulnerability Theory (34) and the Cusp Catastrophe model (35, 36). Klonsky et al. (33) describe suicide as a three-step process: (1) suicidal ideation is generated by a combination of psychological pain and hopelessness, (2) this ideation strengthens when the pain overwhelms feelings of belonging or connection and (3) suicidal ideation transitions to planning and action when the person acquires the capacity for suicide. The psychological pain includes feelings that “one is essentially being punished for engaging with life, which in turn brings a desire to avoid life” (28). When combined with hopelessness about the situation(s), suicidal ideation emerges. The acquired capacity to commit suicide relies on similar factors discussed above including access to lethal means and reduced fear of death. In the FVT model, Rudd (34) focuses on the fact that STBs are a dynamic process that are subject to change over time. Baseline risk factors are relatively stable and differ between people, whereas acute suicidal behavior fluctuates driven by life events and stressors and is time limited. Functional activity across integrated systems – cognitive, affective, physiological and behavioral – determines entry into suicidal mode, the separate action phase of this model. An offshoot of the FVT model, the Cusp Catastrophe Model of suicide also emphasizes the dynamic nature of suicidal behavior and envisions sudden, and typically unpredictable, transitions from low-risk suicide states to high-risk states driven by nonlinear effects of rapid changes (35, 36).

## Limitations of Current Theories of Suicide

The main theories summarized here, along with others [e.g., by Wenzel and Beck (37) and Roberts and Lamont (38)] not discussed (see Table 1), have significant heuristic value and have greatly impacted the field of suicidology. In general, they share common themes and underlying processes: hopelessness/defeat, a sense of burdensomeness, the motivational drive of suicidal ideation and a distinct acquired suicide capability/mode. Herein, lie some of the shortcomings of current theories of suicide. The major limitation is the conceptualization of the ideation/motivation stage and the volition/enaction stage as distinct and separable processes. According to the various models, ideation leads to action in a linear relationship (28); however, these processes are supposedly governed by different sets of factors. Based on the nonlinearity of most complex biological systems, this concept seems unlikely from first principles. Along similar lines, suicidal ideation, suicide attempts and suicide completions are typically considered as distinct entities instead of lying on a spectrum or continuum. In ideation-to-action theories, the intent of someone who considers suicide is often treated as a distinct, but related, state of mind from the intentions of someone who was successful. Moreover, states of mind clearly fluctuate and decisions are constantly questioned and even changed as new developments impinge on the status quo. The FVT model (34) addresses some of these issues, but has limited biological underpinnings. On the whole, theories of suicide inadequately incorporate biological and genetic mechanisms to explain STBs [with some exceptions, e.g., see refs. (43–45)]. For the most part, biology and genetics are considered vague and static distal factors that somehow determine the set point for vulnerability to suicide and are seldom viewed as dynamic processes differentially affecting mental states depending on everchanging conditions. For example, genetic predisposition to conserve effort and ambition when the chance for reward is low (a resiliency factor) may protect against the financial stress of recent job loss, whereas it may exacerbate a later response to social isolation because efforts to engage with others are reduced.

**Table 1:** Summary of notable theories of suicide*.

**Authors**	**Date**	**Brief description**	**References**
Durkheim E	(1897) 1951	Sociological basis of suicide	(39)
Shneidman ES	1985	Psychological pain - psychache	(40)
Rubinstein DH	1986	Stress-diathesis model	(41)
Baumeister RF	1990	Escape theory of suicide	(42)
Mann JJ, et al.	1999	Clinical model of suicide	(29)
Rudd MD	2006	Fluid vulnerability theory (FVT)	(34)
Wenzel A & Beck AT	2008	Cognitive model of stress/suicide	(37)
Van Orden KA, et al.	2010	Interpersonal theory of suicide	(30)
Roberts M & Lamont E	2014	Existentialist reconceptualization	(38)
Klonsky ED & May AM	2015	Three-step theory of suicide	(33)
O'Connor RC & Kirtley OJ	2018	Integrated motivational-volitional model	(31)
Bryan CJ, et al.	2021	Cusp Catastrophe model	(35, 36)

*The table provides a selective historical overview of influential theories of suicide; it is not intended to be comprehensive. The authors and dates listed refer to the corresponding references cited and not to the date associated with the origins of a particular model.

The fact that there appear to be different types of suicide presents a serious challenge to any comprehensive theory of causation. A suicide committed in the depths of relentless depression appears different from that committed by someone diagnosed with a severe or terminal illness and who wants to minimize the burden on their family, and different still from the actions of a “suicide bomber.” In all cases, the outcome is the same, but any similarities in the paths toward that end remain obscure.

## Genetic and Biological Considerations of Risk For Suicide

In the theories discussed so far, genetic factors are considered to be distal or fixed pre-motivational contributors to risk for suicide. Suicidal behavior has a strong familial component (5, 6) and genetic studies confirm strong heritability (7, 8). Consequently, candidate gene analysis and unbiased genome-wide association studies (GWAS) have been conducted to identify risk genes and characterize the genetic basis of suicide. Interesting risk-gene candidates have emerged from this work (12–16): CACNA1C/D (calcium channel subunits), GNAS (G protein subunit alpha S), PDPK1 (3-phosphoinositide-dependent kinase-1), STK33 (serine/threonine kinase-33), HIPK2 (homeodomain interacting protein kinase-2), DCC (netrin 1 receptor) and NTRK2 (neurotrophic receptor tyrosine kinase-2). However, their mechanistic roles in shaping STBs have not yet been established. Moreover, genetic risk has not been integrated very well in most models of suicidal behavior.

Variation in a single risk gene will not cause all of the motivational and suicidal behaviors associated with suicide in man. Perhaps, hundreds of risk genes contribute – each to a small extent – as is the case in most psychiatric disorders (46–49). Therefore, translating genetic variation into the proximal causes of suicide attempts may require investigation of endophenotypes, which are observable traits, behaviors or quantifiable measures mediating a gene's effects on complex disorders/behaviors such as suicide. Ideally, it should be possible to study counterparts of the endophenotypes in animal models (50). Several endophenotypes appear to qualify in this regard: impulsive and aggressive traits, HPA axis response to stress, hopelessness, and serotonergic system dysfunction (18, 19, 50, 51). Here, we propose that diminished motivation states, threat assessment and stay vs. go decisions (resolution of opposing behaviors *via* neural circuitry) should be added to the list of relevant phenotypes. These phenotypes are congruent with the Research Domain Criteria (RDoC) formulation of suicidal behaviors by Glenn et al. (45, 52). The five domains consist of Negative Valence Systems, Positive Valence Systems, Cognitive Systems, Systems for Social Processes and Arousal and Regulatory Systems. Negative Valence Systems include constructs – loss and sustained vs. potential threat (45) – that match threat assessment/existential crisis factors. Positive Valence Systems deal with reward and approach motivation, which corresponds to diminished motivation states, whereas Cognitive Systems reflect constructs such as cognitive control (45), which is involved in selection between opposing behaviors.

Genetic variation will affect gene and protein expression/function, which will in turn affect biological outcomes such as cortisol levels, serotonergic neurotransmission, dopaminergic counter-regulation and relevant neural circuitry. These biological activities mediate responses to stress, levels of impulsivity, motivation to engage in life functions and social behavior. Thus, genes determine the biological substrates of STBs that ebb and flow in response to changes in the environment. Furthermore, environmental factors will interact with genetically-influenced endophenotypes. For instance, a genetic tendency for low motivation to engage with others may lead to acute social isolation when exacerbated by loss of a job with social interactions or a long-distance move away from family. The dynamic nature of suicide risk factors means that biological vulnerability for suicide is person and time specific.

## Catalytic Reaction Model (CRM) of Suicide

The development of a new model of suicidal behavior was motivated by several goals. First, the catalytic reaction model (CRM) reconciles suicidal ideation with subsequent courses of action, and re-emphasizes the dynamic nature of STBs as a spectrum. Second, the CRM incorporates biology and genetics into the theory and to develop ideas that would be testable in animal models. Third, the CRM explains different types of suicide along with the role of environmental factors as contributing conditions for suicide.

**Figure 1:**
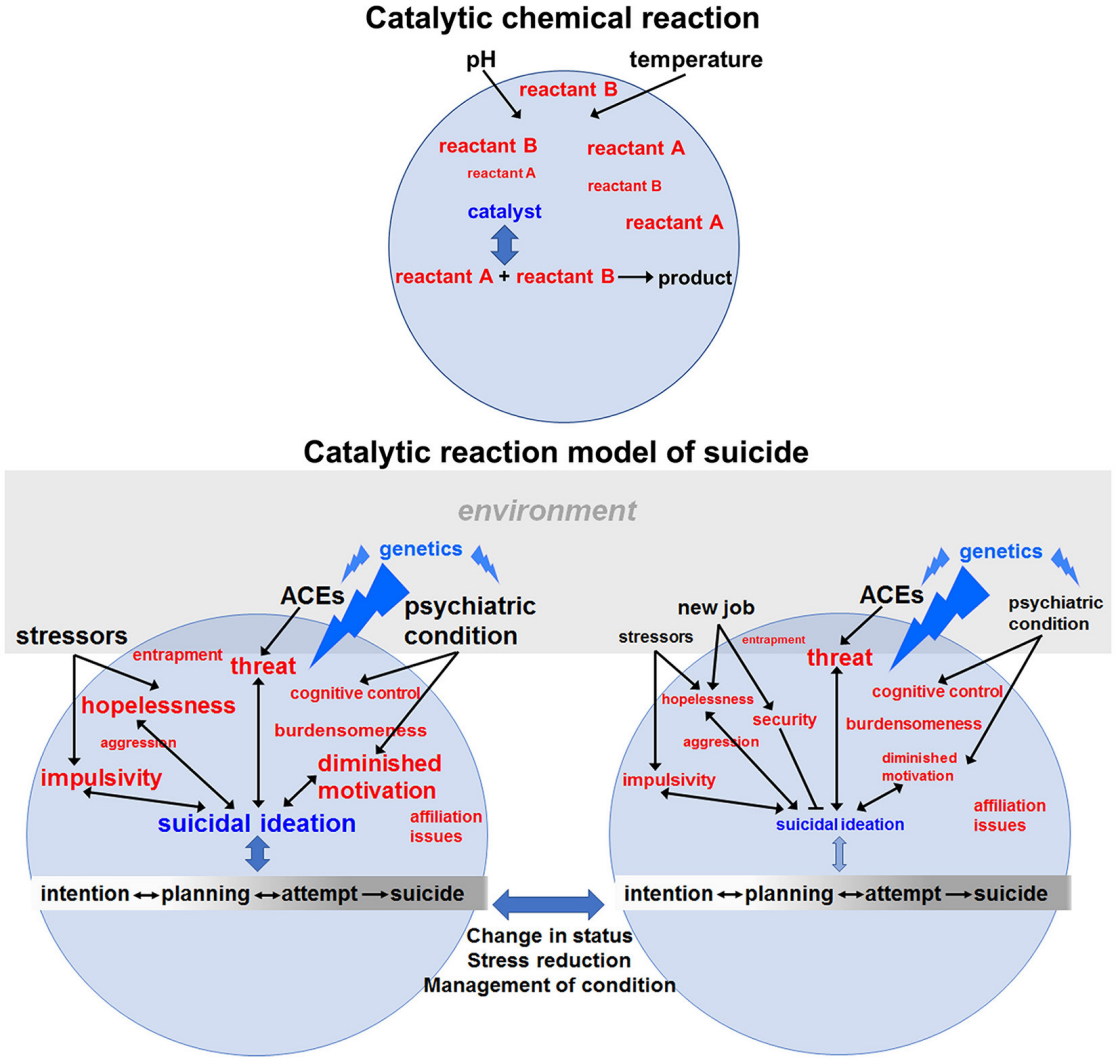
Catalytic reaction model of suicide. In a chemical reaction (upper panel), reactants may combine slowly in the absence of a catalyst, which interacts with both reactants, lowers the energy barrier to the chemical reaction and catalyzes formation of product. Conditions and concentrations of reactants along with the activity of the catalyst determine the rate of the reaction. By analogy, in the lower left panel, various reactants (in red font) combine to determine the catalytic activity of suicidal ideation, which then lowers the “energy” barrier for suicidal behaviors (intention, planning, attempts and completion). The lack of arrows between a pictured reactant and suicidal ideation indicates that at this particular time those factors are manageable and do not contribute to the ongoing suicide reaction. The rate of the suicidal reaction is also determined by environmental conditions (black font) such as stressors or the presence of a psychiatric condition and how well it is being managed. The font size of the reactants and conditions reflects their “concentrations” or “energy” – determined by the intensity, frequency and duration of their effects. Genetic variation (blue font) affects the reaction at several levels by: (1) specifying vulnerability to psychiatric disorders, (2) determining resiliency or acuity in the face of adverse childhood experiences (ACEs) and (3) affecting the emergence and intensity of hopelessness, impulsivity, diminished motivation, etc. in response to changes in the environment. Genetic variants will also affect how a person responds to stressors. As discussed in the next section, genes (e.g., those involved in insulin signaling) affect motivation states, threat assessment (e.g., neuropeptide Y) and impulsivity. In this way, a genetic risk variant can act as a catalyst by lowering the “energy” barrier for expressing various reactants that give rise to STBs. The environment plays a role by affecting motivation (e.g., through reward availability), determining the level of external threat and shaping the context in which opposing behavioral decisions are made. The suicide reaction is dynamic and changes over time, including which risk genes are most important due to differential contributions by different reactants depending on changing circumstances. For example, in the panel at the lower right, the same person represented to the left has now received more effective treatment for their depression and has recently found a new job after a period of unemployment. These changing conditions might lessen feelings of hopelessness, increase a sense of security (a protective factor) and improve motivation to engage in life. Consequently, suicidal ideation is greatly reduced and may be eliminated with continuing progress. Nevertheless, certain levels of impulsivity may remain, and heightened threats may persist due to factors less amenable to change, e.g., ACEs. With lowered concentrations of reactants and a decrease in the catalytic activity of suicidal ideations, residual levels of threat or impulsivity may be tolerable. Suicidal behavior is depicted on a spectrum with arrows showing that some of the processes are reversible. Furthermore, the bidirectional arrows between reactants and suicidal ideation indicate that learned aspects of ideation can feedback to potentially increase feelings of hopelessness, further diminish motivation for enjoyable life activities, etc. The constellation of reactants that drive the reaction will differ between individuals and in relation to their dominance. For instance, a person with a severe debilitating medical condition who contemplates suicide may be primarily driven by feeling like a burden to their family, whereas the ideation of a suicide bomber may be dominated by cognitive control (making disadvantageous choices for political aims) or religious motivations (not depicted). Although two mental states of the individual are represented here, in reality, conditions, reactants and ideation are constantly fluctuating until a crisis is resolved. In our view, prevention should mainly focus on removing threats, promoting engagement in life and mindfulness training to recognize and stop impulsive override of logic. Finally, it will be important to address potential precipitating conditions such as a psychiatric disorder or stressful life events with medications, patient education, coping strategies and therapy as appropriate.

The CRM is based on the conceptualization of suicidal behavior as a type of catalytic reaction (see Figure 1). In a catalyzed reaction, various reactants undergo chemical reactions available to those molecular species. This occurs at much higher rates in the presence of a catalyst. The catalyst typically lowers energy barriers that limit the reaction, thereby accelerating formation of the product, depending on the levels of the reactants and ambient conditions such as pH and temperature. The reaction may be reversible, but the catalyst generally drives it toward completion. Because the catalyst participates in the reaction, it is likewise influenced by the reactants.

Using this analogy, we suggest that multiple “reactants” can combine in a reaction/response that gives rise to suicidal thoughts, which can then serve as a catalyst for suicidal behavior including planning, attempts and suicide (Figure 1). The model encompasses three sets of interacting factors in the suicide reaction: (1) reactants (e.g., hopelessness), (2) external conditions, including environmental impacts such as job loss and personal factors, e.g., adverse childhood experiences (ACEs), and 3) catalysts – suicidal ideation and genetic vulnerability – that lower barriers to action and determine traits/reactants (e.g., burdensomeness or impulsivity) that drive actions, respectively. The reactants in this model are states of mind or behavioral strategies. Suicidal thoughts would represent a kind of catalyst-reactant intermediate whose formation is related to the “concentration” (intensity or severity) of the reactants. In the model, a second potential catalyst for suicide is the individual complement of genetic risk variants that influence the reaction differentially depending on which factors are driving STBs at a given moment. Genetic variation is a catalyst because it can lower the energy barrier (increase vulnerability) to expressing counterproductive behaviors such as hopelessness, feelings of entrapment, etc. or suppress positive coping strategies, thereby activating or accelerating the production of suicidal behaviors. Effects of genetic variation will be mediated through biological substrates of the corresponding behaviors and will include neurotransmitter signaling, neuroendocrine/cytokine function, brain circuitry and more. As reactants shift over time – from loss and anxiety to diminished motivation and hopelessness – genetic variation in different sets of genes will impact the new factors driving STBs. Similarly, environmental factors can potentially modify genetic contributions to the reactants, e.g., *via* epigenetic regulation of genes involved in stress responses and HPA axis hormonal feedback. Environmental factors determine which genes influence the state of mind (reactants) at a given time, whereas genetic factors determine how the person responds to a changing environment. Therefore, genetics is not a fixed contribution to suicide risk. In a fluctuating combination, reactants, catalysts and conditions may generate products in the form of suicide planning (reversible reaction), attempts (quasi-reversible) and completed suicides (irreversible outcome). Whether products emerge will depend on the concentration of reactants and catalysts together with the prevailing conditions at the time, e.g., recent job loss, impending divorce or diagnosis with a terminal medical condition.

The behavioral reaction potentially leading to suicide is dynamic, ongoing (unless interrupted) and subject to fluctuation. If suicidal thoughts are high and certain baseline conditions exist, addition of reactant (e.g., perceived existential threat) or a change in conditions (e.g., social status) can promote completion of the reaction in the form of suicide attempts, regardless of whether or not they are fatal. Conversely, a reduction in reactants (feelings of hopelessness) or a favorable change in conditions (finding a new job) will shift the reaction away from completion (suicide planning and attempts). (Note: in this context, completion refers to halting the progression of planning to prevent a suicide attempt). We envision the entire catalytic reaction as a nonlinear, largely reversible system spanning a continuum from diminished motivation to engage in life to suicidal thoughts and planning, to preparation, attempt and execution. In the model, suicidal thoughts reinforce and magnify detrimental effects of the reactants to lower the barrier or threshold for action. Consequently, ideation and action are not separate, discrete stages, but are connected *via* dynamic interplay until there is resolution – one way or the other.

What are some of the reactants involved in STBs? Unlike standard chemical reactions (Note: our model is an analogy and not a precise mimic), many reactants may potentially participate in the overall response. Hopelessness, defeat, entrapment, perceived existential threats, reduced fear of death and many other reactants may contribute to the development of STBs (29–31, 33). In the model, we focus on several traits/phenotypes in particular to connect with the biology and genetics in the next section; the correspondence of these phenotypes to **RDoCs** (45) has been highlighted in this section. We underscore the importance of diminished motivation to engage in life (**a motivation/reward construct**), exaggerated threat assessment and impulsivity as three significant reactants in the response. A diminished motivation to engage in life would constitute a necessary first step toward suicidal behavior. It roughly equates with anhedonia (a key feature of major depressive disorder) and hopelessness, an established accompaniment for suicidal ideation (29, 53, 54). Exaggerated threat assessment (**sustained or potential threat constructs**) refers to viewing circumstances such as loss of a job, lack of popularity at school or divorce as palpable threats to one's existence. It is the foundation for existential crises and is a contributor to anxiety disorders, which are often comorbid with STBs (38, 55, 56). Finally, impulsivity reflects acting without thinking through the consequences or emotional responses overriding our logical responses (**cognitive control construct**). It has been identified in numerous studies as an important component of suicidal behavior (29, 57, 58).

## Catalysis of STBS by Genetic and Downstream Biological Factors

If genetic risk is a catalyst that modifies the nature and effective “concentration” of reactants such as diminished motivation or threat assessment, it should be possible to connect the two. Here, we summarize connections revealed in studies of the nematode, *C. elegans*, that suggest certain aspects of suicidal behavior may be fundamental to life, evolutionarily conserved and controlled by genetics. Previously, we (59) identified a state of diminished motivation in *C. elegans* regulated by signaling pathways (e.g., Akt) associated with psychosis (60) and major depressive disorder (61). Animals with defects in the insulin receptor gene and downstream signaling components fail to forage in response to food deprivation and will remain in place until they die (59). They are capable of movement during this time, but remain largely immobile, which is reminiscent of the response of mice and rats in the forced swim test – a rodent model of depression (62). This response was compared to suicidal behavior because the diminished motivation state was fully corrected with antidepressant drugs and clozapine (63), established treatments used in suicide prevention (64–68). Diminished motivation to search for food results from imbalances in serotonergic and cholinergic function (59). Recently, animals with defects in genes implicated as risk factors for suicide (including orthologs of STK33, HIPK2 and DCC mentioned above) showed the same diminished motivation phenotype that was also corrected with antidepressants and clozapine (D.S.D., unpublished observations). These risk factors have not been thoroughly characterized in humans and their roles are likely to be complex. We have already gained a relevant foothold in *C. elegans*. Moreover, the relative simplicity of this system may allow us to establish mechanistic connections to counterparts of human suicidal behavior that would otherwise be overlooked.

At first glance, it may seem farfetched to consider the foraging response of *C. elegans* informative with respect to motivation to commit suicide. However, all animals must acquire food to live, and by not engaging in foraging the mutant strains are not engaging in life. It would be fitting that investigation of a behavior necessary to sustain life might also provide insight into self-inflicted behaviors to end it.

*C. elegans* must assess and respond to threats in the environment. Genetic mutations can produce an overly keen sense of threat in response to perceived levels of ambient O_2_ that causes animals to aggregate or “social feed” on bacterial lawns (69, 70). This phenotype is prominent in strains with defects in the neuropeptide Y receptor (*npr-1*) gene (69). This receptor signaling pathway is involved in anxiety in rodents and man (71–73) and has been identified as a risk factor for suicide (23–25). *C. elegans* strains with loss-of-function mutations in the 3-phosphoinositide dependent kinase-1 (*pdk-1*, mentioned earlier) also show aggregation (social feeding) that is corrected with clozapine and lithium (D.S.D., unpublished observation); the latter drug is also effective in decreasing suicidal behavior (74–76). Consequently, we wonder if exaggerated threat assessment in man may be regulated in a similar way. An exaggerated threat response would allow common events such as changes in social status to be perceived as existential threats and crises. By studying the genetics and mechanisms that cause exaggerated threat assessment in *C. elegans*, we may obtain insights into similar processes in persons with STBs.

The last reactant that will be discussed here is impulsivity. Impulsivity sits at the crossroads of logic and emotion. It is involved in the cognitive control of stay vs. go decisions that select between psychomotor programs specifying incompatible and/or opposing actions. An animal can stay and eat or go and forage, but cannot do both at once. Similarly, a person can be logical and analytical or emotional and impulsive, but cannot be both simultaneously. Brain circuitry controls which of the opposing actions will be selected and implemented. In *C. elegans*, we identified such a circuit, called a counter-circuit, involving two sets of dopaminergic neurons that control opposing actions (63). The sets of neurons receive different inputs and send collateral processes to regulate the other pathway. The dopaminergic neurons bear D2-type dopamine receptors such that when one dopaminergic pathway is active, it releases dopamine that suppresses the other pathway *via* the D2 receptors, thus preventing the opposing action. We suggest that impulsivity and logic are regulated by similar circuitry in humans (63) and in fact, dopaminergic projections to the limbic system and frontal cortex appear to have a counter-circuit arrangement involving collateral processes and D2 receptors (77–79). According to the model, impulsivity created by an excess of limbic activity (an overly simplistic view) causes opposition override of logical input from the frontal cortex in a counter-circuit. Finally, religious beliefs or strong ideology can perform the same function *via* a counter-circuit and override opposition (e.g., fear of death or moral objections) to becoming a suicide bomber.

The fundamental behaviors discussed in this section overlap with some of the endophenotypes for STBs proposed by others, namely hopelessness, serotonergic dysfunction and impulsivity (50, 51). Therefore, basic components of suicidal ideation and behavior are evolutionarily conserved, which means animal models may provide useful insights even if they fail to fully recapitulate suicide. Moreover, the work in *C. elegans* shows mechanistically how variation in suicide risk genes can produce endophenotypes such as diminished motivation that are potentially relevant for suicidal behavior.

## Advantages and Limitations of the Model

The main advantage of the catalytic reaction model is that it integrates what is known about the genetics and brain circuitry of suicidal behavior with established risk factors including hopelessness (diminished motivation), existential crises (exaggerated threat assessment) and impulsivity (opposition override) to provide biological foundations and/or explanations for STBs. By conceiving of suicide as a semi-reversible catalytic reaction where suicidal thoughts and genetic risk catalyze planning, attempts and suicide, the model reflects a dynamic continuum of behaviors rather than discrete stages affected by different factors. Moreover, the concept of multiple factors (reactants) contributing to the overall reaction negates the need to ascribe single or limited sets of reactants (e.g., hopelessness or thwarted belongingness) as the most important causative factors to the exclusion of others. Suicidal behavior is a changeable process anyway, so the greatest concerns and driving forces during early stages of developing suicidal ideation may be different from the major factors that tilt the balance toward action at a later time. The reversible nature of the reaction can also explain why only a fraction of those with suicidal ideation actually commit suicide. Although suicidal thoughts are an effective catalyst for suicide, the reactants and response conditions (altered socioeconomic status, decline in general health, ACEs, etc.) must together achieve a critical mass for the reaction to proceed.

The theory includes many testable components and encourages exploration of how genes affect phenotypes representing fundamental behaviors that normally sustain life. It also can account for gene-environment interactions that unfold as conditions change or new reactants arise. Finally, although suicidal ideation emerges from thoughts of hopelessness or inescapable harm, override of opposition (e.g., survival instincts or moral compunction) is an important feature of the model and may be mediated by impulsivity or strong ideological motivation. For example, a suicide bomber may perceive an outside, existential threat to their way of life that can only be met with extreme self-sacrifice.

Every theory of suicide potentially advances our knowledge, but there may be limitations too. The present model is wide in scope and highlights dynamic aspects of the process (similar to the FVT), which is attractive from a descriptive standpoint, but makes it more difficult to delineate the precise role of the reactants. Another potential limitation concerns the best way to narrow focus on those factors that will allow accurate prediction of suicidal intent in order to initiate prevention strategies. We have spotlighted several of what we view to be the most salient factors here; however, these are subject to bias. It is also plausible that suicide is inherently a chaotic process reflecting the state of mind of someone considering it, and this process, by definition, may resist full characterization and elude predictability. Chaotic processes contribute to the liability for psychiatric disorders (80), therefore this possibility merits serious consideration. Any model of suicide will be subject to this last limitation.

## Environmental Conditions of the Reaction: Stress and Related Factors

The model suggests that stressors contribute significantly to the conditions that determine suicidal reactions. Two aspects will be covered here: (1) the general role of stress and (2) a specific example drawn from ongoing events – the COVID-19 pandemic. Stressors such as psychiatric disorders and challenging life events (e.g., medical illness or divorce) are important risk factors for suicide (81). Psychosocial crises and psychiatric disorders may constitute the stress component of the stress-diathesis models of suicidal behavior (29). The exposure to repeated acts of abuse and other adverse childhood experiences significantly increases risk of suicidal behavior throughout a person's life (82).

The involvement of stress in suicide permeates all the way to the molecular level. Maternal deprivation in infant rats causes changes in DNA methylation and expression of glucocorticoid receptor genes, leading to impaired feedback inhibition and ultimately elevated release of cortisol during an overactive stress response in adults (83, 84). Cortisol is the primary effector hormone of the HPA axis stress response system. Blunted cortisol responsiveness to stress (low baseline levels) is associated with suicide attempt in adults (85), possibly reflecting an adaptive response in the stress system. In addition, a lower baseline level of cortisol was identified as a potential trait that confers vulnerability to suicidal behavior (86, 87). By contrast, Giletta et al. (88) found that heightened cortisol reactivity during a psychosocial stress task was the strongest predictor of suicidal ideation at 3-month follow-up in at-risk adolescent females. This suggests that biological mediators of the stress response impact suicide risk and may augment the effects of exaggerated threat assessment and diminished motivation.

Current evidence supports the notion that vulnerability to STBs is continuous. Each time suicidal behavior is activated, it becomes increasingly accessible in memory and requires fewer triggering stimuli to become activated the next time (89). This learned component of suicidal behavior may help to explain why people differ in their reactions to similar stressful life events spanning from disappointment and depression to deliberate self-harm and completed suicide (89).

In view of these connections between stress and suicide, the recent emergence of COVID-19, a life-threatening and chronic stressor, is cause for concern. Already, COVID-19 has been shown to adversely affect mental health by generating reactants such as loneliness and hopelessness with the rise in the number of cases (90–92). Additionally, the implementation of lockdown and business closings have caused social isolation and feelings of disconnection, which have exacerbated pre-existing mental health issues (92, 93). Social isolation has been associated with increased loneliness, anxiety, depression and early death (94). Moreover, social isolation exacerbates suicidal ideations, causing a detachment from support systems along with an increased risk of suicide (95, 96). The COVID-19 pandemic has also caused financial stress, which can create hopelessness, lower self-worth and increase mental health problems including suicidal ideation – the major catalyst in our model (96–98).

A recent US survey found that 45% of adults reported COVID-19 had caused immense worry and stress and negatively impacted their overall mental health (91). Furthermore, the pandemic has been linked to increased levels of substance use (90). Together, these COVID-19-related stressors increased suicidal ideation, suicide attempts and completed suicides (99–102). These observations held true for most age groups. The Coronavirus certainly qualifies as an existential threat, consistent with our model. Moreover, the resultant lockdowns and social distancing unintentionally spawned reactants in the form of social isolation, hopelessness and diminished engagement in enjoyable life activities. Thus, a major effect of COVID-19 and its associated stressors has been to increase the concentrations of catalysts and reactants available to promote an untoward reaction.

## Implications for Prevention of Suicide and Treatment Of STBS

The view of how STBs manifest and evolve will affect strategies aimed at prevention of suicide and treatment of at-risk individuals. Accurate prediction of who is likely to attempt suicide is both a major challenge and key to prevention (4). The objective is to ascertain where a person with suicidal ideation stands in the reaction scheme proposed here, i.e., are there sufficient catalysts and reactants to drive a behavioral response toward attempted or completed suicide? Modifiable factors include the presence of a psychiatric condition, substance use, hopelessness, aggressive/impulsive tendencies, isolation/loneliness, loss, and an underlying medical condition (43, 103–105). In addition, cultural and religious beliefs may support the notion that suicide is acceptable or respected (106, 107). Non-modifiable “conditions” include male gender, older age (especially with infirmity), white or native American race, history of ACEs, and suicide in a family member or close friend (43, 103–105). Recent or current hospitalizations are a strong risk factor for suicide (108); however, most suicides are completed by individuals who have not been hospitalized (104). Consequently, numerous factors must be evaluated and altered to effectively interrupt the suicidal reaction.

Previous work has suggested the following approaches could be used to prevent suicide: education programs for the general public and professionals, treatment of existing psychiatric conditions, changes to media reporting of suicide, restriction to access of lethal means, and screening methods, especially for those at high risk (109, 110). Meta-analyses support the efficacy of restricted access to lethal means, education programs for physicians and school-age children and cognitive behavioral therapy [CBT; (109, 110)]. Moreover, there is evidence in adolescents to suggest that multilevel prevention programs can help prevent suicide. The Nuremberg Alliance Against Depression is the best-evaluated intervention and included cooperation with primary care providers, a professional public relations campaign, training community facilitators, and self-help groups. This intervention resulted in a 24% reduction in suicidal acts (111). Altogether, these prevention strategies will increase awareness of risk factors for suicide, reduce the severity of reactants, such as hopelessness and isolation, and increase the barrier to suicide.

When evaluating a patient for STBs, it is critical to establish whether there is a cogent plan along with access to lethal means or a history of previous attempts and recent disengagement in life activities such as interactions with family and friends. Upon completing of the evaluation, the clinician should gauge the patient's disposition and decide whether hospitalization is necessary, and effectively address any existing psychiatric conditions. Finally, stressors should be targeted, triggers of STBs should be avoided, and family and friends should be involved in the process.

From a pharmacological standpoint, studies support the use of clozapine and lithium to reduce suicide risk in appropriately targeted populations (66, 74–76, 112). For example, clozapine significantly decreased suicide attempts in patients with schizophrenia and schizoaffective disorder (66, 112). In addition, lithium reduced the risk of suicide in patients with bipolar disorder and major depression (74–76). In a previous section, we discussed how these drugs modify behavioral counterparts of STBs in model organisms. Antidepressant drugs have a role in addressing symptoms of depression in at-risk individuals; however, they offer less benefit in suicide prevention (110). In fact, some studies have shown antidepressants may increase STBs especially in children, adolescents and young adults (113–115). We suggest that clozapine and lithium may address the neurobiological processes underlying overactive threat assessment, diminished motivation to engage in life and selection between opposing behaviors.

## Conclusions

The CRM offers a novel view of suicide and the driving forces behind it. Because many different reactants that vary in intensity, frequency and duration contribute to STBs along with changing environmental conditions, it should be possible to interrupt suicidal behavior at different points and with different strategies. The overall approach would be to change reaction conditions and the energy/intensity of reactants to reduce the catalytic activity of suicidal ideation. A primary goal would be to train individuals with STBs to self-monitor and identify states of mind in order to regulate reactants (especially impulsivity/emotionality and symptoms) and restore low catalytic activity and safety. At the same time, mindfulness and cognitive behavioral skills (including dialectical behavioral therapy [DBT] and CBT for suicide prevention), aimed at identifying cognitive distortions, can enhance appreciation that perceived threats can be effectively managed. Furthermore, if individuals with suicidal ideation are introduced to the concept that suicidal behavior is a dynamic process rather than a predictable path or inevitable solution, they may be more willing to collaborate in developing a treatment plan to address the conditions and reactants and ultimately exert control over the catalyst. Encouraging greater engagement in life activities and involvement in social interactions or advancing the greater good of society will instill positive goal-directed behavior that redirects from suicidal ideation. Through the CRM process, current status and likelihood for action would be evaluated, while affording the person agency to project themselves into the future in the context of both positive and negative changes in reactants and conditions and to master these forces. In therapy, the CRM process would promote extrapolating behavior to visualize new life trajectories, self-monitoring and the use of regulating skills leading to the inception of hope. It is important that the person understands that reactants will not completely disappear, but can be reduced and managed with psychoeducation, learning and practice, which builds self-esteem and self-efficacy. Collectively, these prevention strategies would decrease the catalytic activity of suicidal ideation, raise the energy barrier to suicidal behavior and provide alternative reaction pathways with positive outcomes.

## Author Contributions

DD conceived of the topic and major ideas, wrote, and edited the paper. PM, SS, AS, and WT contributed to the writing, editing, and revision of the manuscript. All authors contributed to the article and approved the submitted version.

## Conflict of Interest

The authors declare that the research was conducted in the absence of any commercial or financial relationships that could be construed as a potential conflict of interest.

## Publisher's Note

All claims expressed in this article are solely those of the authors and do not necessarily represent those of their affiliated organizations, or those of the publisher, the editors and the reviewers. Any product that may be evaluated in this article, or claim that may be made by its manufacturer, is not guaranteed or endorsed by the publisher.
